# *Beauveria bassiana* Induces Strong Defense and Increases Resistance in Tomato to *Bemisia tabaci*

**DOI:** 10.3390/jof11020141

**Published:** 2025-02-13

**Authors:** Mengying Liu, Dong Xiang, Heikki M. T. Hokkanen, Tiandi Niu, Junjie Zhang, Jinlin Yang, Qiuyang Wei, Hanqiu Chen, Huai Liu, Yaying Li

**Affiliations:** 1 Yibin Academy of Southwest University, Southwest University, Yibin 644000, China; liumengying@swu.edu.cn (M.L.); ysdtz1912@email.swu.edu.cn (T.N.); zhangjunjie627@163.com (J.Z.); xzlpsyjl@163.com (J.Y.); weiqy_88@126.com (Q.W.); 2 Key Laboratory of Agricultural Biosafety and Green Production of Upper Yangtze River, College of Plant Protection, Southwest University, Chongqing 400715, China; 3 Institute of Vegetable, Tibet Academy of Agriculture and Animal Husbandry Sciences, Lhasa 850032, China; xiangd666@126.com (D.X.); chenhanqiu11@126.com (H.C.); 4 Stockbridge School of Agriculture, University of Massachusetts, Amherst, MA 01003, USA; heikki.hokkanen@ecostack.eu

**Keywords:** phenylalanine deaminase, induced resistance, phenolics, antioxidant

## Abstract

Pre-stimulation of plants can change their resistance mechanisms, thereby enhancing their defense responses. *Beauveria bassiana*, a broad-spectrum entomogenous fungi, can also induce plant defenses, but it received little attention. Here, we show that *B. bassiana* can act as a stimulus to prime tomato defense responses, improving resistance in the plant to herbivore stress. The results illustrated that four defense genes (*PIN2*, *PR2*, *PAL*, and *MPK3*) were upregulated in all *B. bassiana* treatments, especially the phenylalanine deaminase (*PAL*) gene, which was highly expressed in tomato plants after *B. bassiana* inoculation. Feeding through *Bemisia tabaci* resulted in a weak upregulation of defense genes. However, in combined fungal inoculation and *B. tabaci* feeding, a total of nine defense genes were upregulated, among which five genes—*PAL*, *PPO*, *PIN2*, *PR2*, and *PR1*—were closely related to the phenol synthesis. The results of tomato plant metabolism showed that *B. bassiana* mainly activates tomato phenylpropane metabolic pathways, with this modulation being influenced by jasmonate. Further explorations revealed a significant enhancement in the antioxidant capacity of the plants, as evidenced by the determination of their antioxidant compounds and the coloration of leaf phenolic substances. Thus, entomopathogenic fungi can act as an exogenous substance to activate the defense responses of tomatoes without damaging the plant, indicating a good potential for developing applications using *B. bassiana* to promote resistance in tomatoes for pest management.

## 1. Introduction

Plants are exposed to a wide range of pests and pathogens to which they respond by activating a variety of defense signaling pathways [1]. The defense responses constitute localized responses that reduce the outside attack at the sites of stress, as well as systemic responses that impart resistance to other parts of the plant on a subsequent attack [2]. This inducible defense could be activated by some beneficial microbes, such as rhizosphere microorganisms and entomogenous fungi [3].

*Beauveria bassiana* is an entomogenous fungus widely used in biological control. The role of pathogenic mechanisms in insect infection has been described [4], but previous studies have predominantly focused on pest pathogenicity, ignoring the interactions among plants, insect-pathogenic microorganisms, and pests. Microbes are not only associated with insects but also can profoundly influence plant–insect interactions [5,6,7]. Indeed, it has been confirmed that entomogenous fungi *Verticillium lecanii* and *Metarhizium anisopliae* could stimulate plants by altering plant metabolic components, induce attraction or repulsion to insects, and may be toxic to pests [8,9,10].

Plants were subject to feeding stress by many pests, evolving a series of complex defense systems against insect herbivores, such as *Bemisia tabaci* [11]. Researches showed that *B. tabaci*, a generalist herbivore, damages crops by feeding on phloem sap and may suppress induced plant responses, including reducing the induction of the jasmonic acid (JA)-mediated defense signaling pathway via its salivary protein, the ferritin BtFer1 [12,13]. Therefore, the importance of enhancing plant defenses and eliminating pest interference effects is indisputable.

Since the publication of the review article ‘Priming: getting ready for battle’, the importance of defense priming as an adaptive trait for the adjustment of plant defense in unpredictable environments has been well established [14]. Defense priming has been proposed as an adaptive and cost-effective defensive strategy, characterized by a subtle and transient response initiated by specific stimuli. In primed plants, defense responses are activated more rapidly, robustly, or sustainably, enabling them to better address challenges compared to unprimed plants [15,16]. Moreover, much of this type of resistance can be passed down through generations [14]. Defense priming can be triggered by various factors, including pest herbivores, pathogens, and chemical compounds, as well as beneficial edaphons, rhizosphere microorganisms, and even entomogenous fungi [17,18].

Plant stimulation can enhance their resistance by eliciting a stronger defense response, serving as an adaptive mechanism to cope with external stress. Defensive traits may encompass a range of alterations, including changes in defense-related processes, signaling compounds, and actual defense responses [19,20,21]. Secondary metabolites in plants are compounds synthesized by plants themselves without influencing normal plant growth and development, serving as direct indicators of plant defense functions [22]. Among the secondary metabolites, phenolic substances are among the most prevalent and widely distributed components, playing a crucial role in enhancing plant defense mechanisms [23,24]. When plants respond to insect feeding attacks, the phenylpropane metabolic pathways are activated, mediating the production of phenolic substances, and subsequently, the content of reactive oxygen species increases to resist pests [25,26].

Jasmonic acid (JA) is a central regulator of plant defense responses and is a lipid-derived compound commonly found in plants, belonging to the oxylipin family of lipoxygenase products [27,28]. It can be obtained from α-linolenic acid as a substrate through the catalysis of lipoxygenase (LOX) and β-oxidation reaction [29,30]. JA can respond to pathogen infection or herbivore feeding, thereby activating the expression of defense genes such as pathogenesis-related genes (PRs), LOXs, and vegetative storage proteins (VSPs), and simultaneously regulating the increase in the activities of defense enzymes such as protease inhibitor (PI), polyphenol oxidase (PPO), and catalase (CAT), playing a crucial role in plant growth, development, and defense [31,32].

Some studies indicate systemic protection of *Papaver somniferum* against *Iraella luteipes* by an endophytic strain of *B. bassiana* [33]. Entomogenous fungi can colonize corn, weakening the drill collar activity of lepidopteran larvae on stalks [34]. These findings suggest that entomopathogenic fungi can act as inducers to enhance plant defense, but research on the mechanisms of enhancement is incomplete.

The aim of this study was to check the hypothesis that *B. bassiana* can stimulate the plant to release a faster and stronger response to *B. tabaci* stress. To assess this, quantitative real-time PCR and gas chromatograph mass spectrometry were used to detect plant primary defense pathways induced by *B. bassiana*, and plant antioxidant tests were performed to evaluate the plant’s defensive ability. This study contributes to our understanding of how *B. bassiana* continually interacts with plant defenses and enhances plant resistance to the generalist herbivore *B. tabaci*.

## 2. Materials and Methods

### 2.1. Insect, Fungal, and Plant Materials

The insect used in the experiments was the whitefly *Bemisia tabaci* (biotype: MED) adults, which were collected from vegetables in the Chongqing Tongnan district, China, and maintained at 26 ± 1 °C, relative humidity (RH) 75 ± 5%, and light (L):dark (D) = 14:10, using tomato (*Solanum lycopersicum*) as a host plant for more than 30 generations. The fungal strain was *Beauveria bassiana* Bb252, stored in the Biotechnology Center of Southwest University at −80 °C. It was originally isolated from *Chilo suppressalis* on maize in Yongchuan District, Chongqing, China, and was separated into a monosporic culture. The fungus was grown on potato dextrose agar (PDA) (pH = 6.5–6.7) at 28 ± 1 °C in a temperature-controlled chamber under darkness. Tomato seeds were purchased from Shanghai Changzhong Tomato Co., Ltd., Shanghai, China, without germination tests. Before sowing, they were sterilized by soaking in 1% NaClO for 5 min, then rinsed three times with sterile distilled water [35,36]. The seedlings were then planted in sterilized nutrient soils (dry sterilization: 75 °C for 24 h) and transferred to an artificial climate chamber at 26 ± 1 °C, RH = 75 ± 5%, and L:D = 14:10. The test started when the tomato plants reached the height of 20 cm.

### 2.2. Experimental Design

There were a total of five treatments in this study. Five plant treatments were included: (1) plants were foliar sprayed with 10 mL of 1 × 10^8^ conidia/mL concentration of spore suspension and tested after 3 days; (2) plants were foliar sprayed with 10 mL of 1 × 10^8^ conidia/mL concentration of spore suspension and tested after 7 days; (3) plants were foliar sprayed with 10 mL of 1 × 10^8^ conidia/mL concentration of spore suspension and tested after 14 days; (4) whiteflies were released on plants at a density of 30 adults per plant to feed for 7 days; (5) fungal inoculation consistent with treatment (2), combined with inoculation with *B. tabaci* 7 days later, when 30 whitefly adults were released for per plant and were allowed to feed for 7 days before test [20,37]; and (6) plants spraying with 0.1 mL of methyl jasmonate (C_13_H_20_O_3_ assay ≥ 95% Coolaber Science & Technology, Beijing, China). Each group contained four replicates. The subsequent experimental treatments were all numbered accordingly.

### 2.3. Tomato Plant Defense Gene Amplification and Characterization

Total RNA from *Solanum lycopersicum* was isolated from fresh lamina tissue using pBIOZOL Plant Total RNA Extraction Reagent (BIOER Technology Co., Ltd., Hangzhou, China). The RNA concentration and purity were determined using a NanoDrop^TM^ Spectrophotometer ND-2000 (Thermo Scientific, Waltham, MA, USA). Rapid amplification of cDNA with the PrimeScript™ RT reagent Kit (TaKaRa, Otsu-shi, Japan) was performed according to the manufacturer’s instructions. Based on the RNA-seq data, the transcript abundance of multiple defense pathway key genes was quantified. The primers used in this experiment are listed in Table S1. Regarding gene expression, a two-step method was used for quantitative real-time PCR detection with the following thermal cycling conditions: 95 °C 1 min, followed by 40 cycles of 20 s at 95 °C, and 1 min at 60 °C. All reactions were run in duplicate technical replicates, and average values were used in the subsequent analysis. The gene expression was quantified by using the 2^−ΔΔCt^ method with *β-actin* as an endogenous control gene and by drawing a heat map based on genes’ relative expression multiples. In this experiment, the treatments (1), (2), (3), (4), and (5) and a control group (plants without treatment) were used.

### 2.4. Plant Metabolite Extraction

In this experiment, the treatments (2), (4), and (5) and a control group were used. Fresh tomato leaf samples (1 g) were initially freeze-dried at −50 °C for 48 h to remove moisture, then ground and placed into headspace vials with 1 mL acetone (HPLC) for 24 h [38,39]. The obtained extracts were filtered through a 0.2 mm polytetrafluoroethylene (PTFE) filter to eliminate impurities. The supernatant fractions were collected and stored at −20 °C (not more than 2 days) until used. The samples were evaporated in a vacuum freeze dryer and derivatized with 200 μL N, O-Bis (trimethylsilyl) trifluoroacetamide (BSTFA) prior to GC–MS analysis [40].

### 2.5. GC–MS Analysis

In this experiment, the treatments (2), (4), and (5) and control group were used. The samples were analyzed using an Agilent 7890B GC-MS system (Agilent Technologies, Santa Clara, CA, USA) equipped with a DB-5MS fused-silica capillary column (30 m × 0.25 mm, 0.25 μm). Helium (grade 5.0, 1.5 mL min^−1^) was used as carrier gas. The injector was operated in the splitless mode for all chromatographic runs. The extracted sample using the M/C technique was analyzed under the following oven temperature program: injector temperature was 250 °C, 35 °C for 1 min, followed by 2 °C/min oven temperature ramp to 70 °C, then by 5 °C/min to 250 °C, and concluding with a 24 min isothermal hold at 280 °C. Mass spectra were acquired using the full scan monitoring mode with a mass scan range of 40–650 *m*/*z*. Qualitative analysis was performed by comparing the obtained MS spectra with the NIST library and ChEBI library. Plant metabolites were annotated for pathways through the Kyoto encyclopedia of genes and genomes (KEGG) pathway. The MetaboAnalyst 6.0 tool (https://www.metaboanalyst.ca/, 20 December 2024) was employed to conduct pathway enrichment analysis on the mass spectrometry results.

### 2.6. Plant Total Antioxidant Activity

In this experiment, the treatments (2), (4), (5), and (6) and a control group were used. The tomato plant samples were prepared using 0.5 g of fresh plant tissue homogenized in 2 mL of sterile water. The total antioxidant capacity was determined using a T-AOC Assay Kit (Nanjing Jiancheng Bioengineering Institute, Nanjing, China, A015-2-1). According to the kit instructions, serially dilute the standard Trolox (an antioxidant), then add the ABTS working solution and let it react for 6 min. Read the OD_405_ and fit the standard curve. Subsequently, in a similar manner, replace the standard with the sample to be tested, add it to the ABTS working solution, react for 6 min, and read the OD_405_. Calculate the Trolox-Equivalent Antioxidant Capacity according to the standard curve.

### 2.7. Phenolic Substance Staining

In this experiment, the treatments (4) and (5) and a control group were used. A 0.5% toluidine blue staining was used for the phenolic substance of plant leaf staining via the characteristic that phenolic substances can combine with dyes and produce distinct blue-purple colors. Wash the leaves with PBS buffer 3 times, add the dye dilution 200 times, and dye for 30 min. Then, wash the leaves twice with PBS buffer and take pictures using an Olympus optical microscope (Olympus Corporation, Tokyo, Japan).

### 2.8. Statistical Analysis

All data are presented as mean ± SE. Statistical analysis was performed using ANOVA and Tukey’s test, with significance reported when *p* < 0.05. IBM SPSS Statistics V21.0 software was used for all data calculations, and the figures were produced using GraphPad Prism 8.

## 3. Results

### 3.1. Tomato Plant Defense Gene Expression

**Figure 1:**
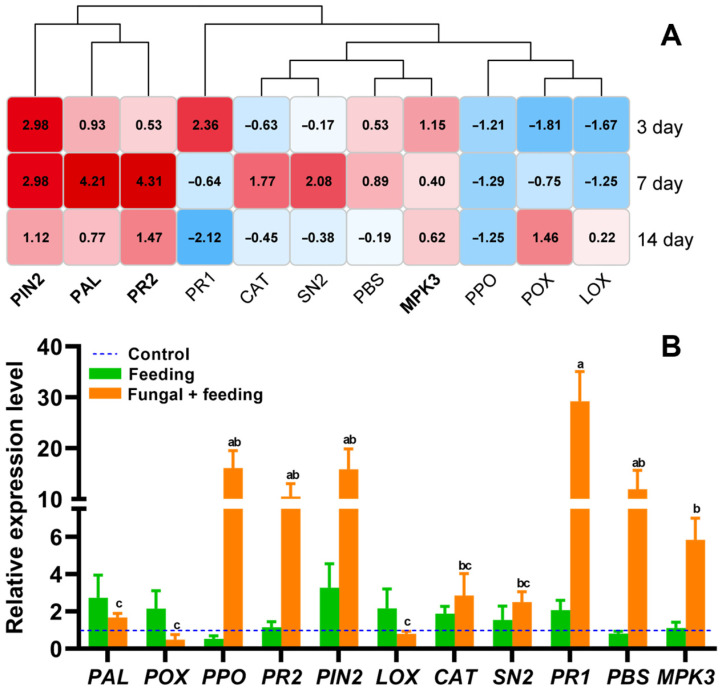
The expression patterns of defense genes in tomato. (**A**) Red represents upregulated expression; blue represents downregulated expression. The numbers in the figure represent the differential expression levels (the fold change) of various genes compared with the control. The bolded genes in the figure refer to genes that were upregulated for expression at all three times. (**B**) Feeding means only feeding by *B. tabaci*; fungal + feeding means *B. bassiana* treatment combined with *B. tabaci* feeding. The bars in the figure show the expression levels of each gene after different treatments. Different letters indicate significant differences (Tukey’s multiple comparison test).

Tomatoes rely on a complex defense system, and the expression of defense genes can be conveniently detected by quantitative PCR. The expression of defense genes in tomatoes was significantly upregulated after 7 days of *B. bassiana* treatment. Compared with 3 or 14 days of treatment, the number of upregulated defense genes at this time was the largest, and the upregulation fold was higher, showing a higher defense level. Four genes (*PIN2*, *PR2*, *PAL*, and *MPK3*) were concurrently upregulated at all three time points, with the phenylalanine deaminase gene (*PAL*) showing particularly high expression in tomato plants following *B. bassiana* inoculation. Its expression reached an 18.5-fold level with a peak at 7 days after the fungal treatment (F_10,43_ = 8.181, *p* < 0.001, Figure 1A).

Compared with fungal inoculation, *B. tabaci* feeding induced gene expression at a lower level, and only a few defense genes were upregulated, including *PAL*, *POX*, *PIN2*, *LOX*, *CAT*, and *PR1*. The average gene expression level was two-fold. However, the gene expression profiles in plants with combined fungal inoculation and insect feeding produced remarkable results: 9 genes were upregulated, and the increases were over 10-fold (F_10,43_ = 3.566, *p* = 0.003, Figure 1B). In this case, defense response genes were mainly in the primary and secondary metabolic pathways, of which four upregulated genes (*PPO*, *PIN2*, *PR2*, and *PR1*) were identified as being involved in phenolic compound synthesis.

### 3.2. Identification of Plant Metabolites

**Figure 2:**
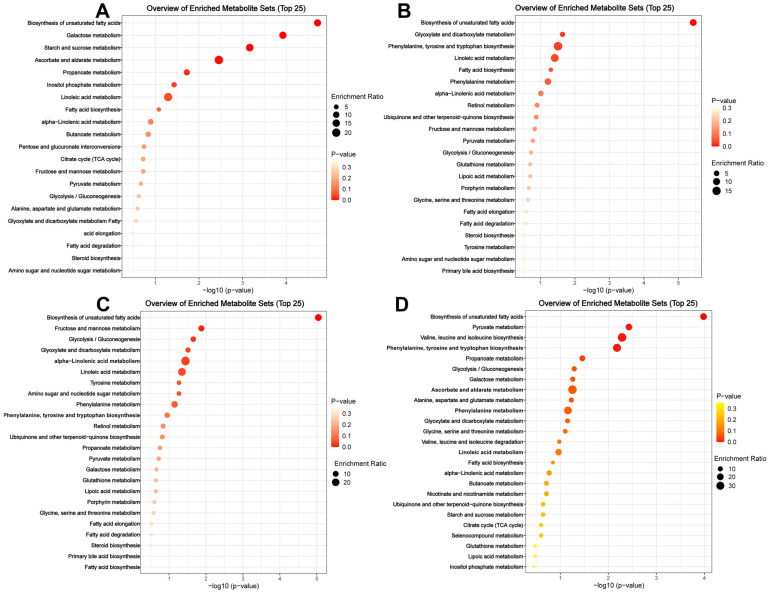
Kyoto encyclopedia of genes and genomes (KEGG) pathway enrichment analysis. (**A**) gives results for the control; (**B**) gives results by only spraying *B. bassiana*; (**C**) gives results for *B. tabaci* feeding; and (**D**) gives results for *B. bassiana* treatment combined with *B. tabaci* feeding.

The GC–MS results for the four treatment groups were different. The mass spectrometry results were compared based on the KEGG Pathway database. The results showed that the plants always take nutrient anabolism as the primary approach (Figure 2). The nutritional pathways obtained by comparing metabolites focus on fatty acid and amino acid synthesis and gluconeogenesis.

**Figure 3:**
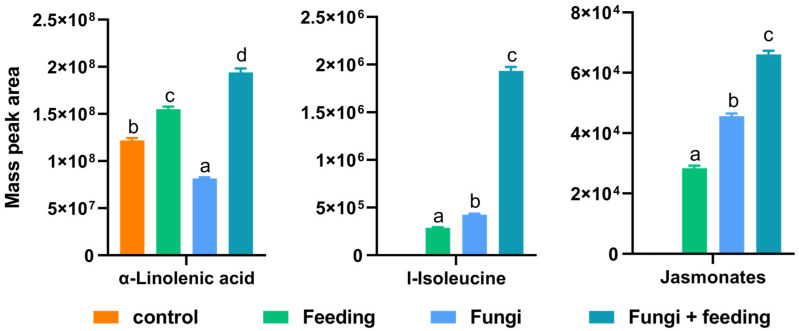
The relative content of the jasmonates and their precursors. The peak area of the mass spectrum was used to indicate the relative content of the substance. Different letters indicate significant differences (*p* < 0.05; Tukey’s multiple comparison test).

Specific defense metabolites were produced only after being subjected to external stress signals. The plant secondary metabolite compounds increased sharply in *B. bassiana*-induced plants. Through compound database comparison, these belong to defense pathways and regulation of the hormone network, such as phenylalanine metabolism, linoleic acid metabolism, and alpha-linolenic acid metabolism (Figure 2B). Treatment with feeding by *B. tabaci* also induces phenylalanine metabolism, alpha-linolenic acid metabolism, glycolysis/gluconeogenesis, and glyoxylate and dicarboxylate metabolism (Figure 2C). Particularly striking was the result that the treatment combining fungal inoculation with insect feeding resulted in the activation of more defense-related pathways, with phenylpropaine metabolism mediated by *PAL* being abundant and prominent. In addition, phenylalanine, a crucial precursor involved in the synthesis of defensive substances such as phenols, had its biosynthetic pathway significantly enriched (Figure 2D). Furthermore, the phytohormone involved in plant defense regulation, jasmonate, along with its synthetic precursors, α-linolenic acid, and I-isoleucine, were detected. For the normalized peak-area calculations, their contents slightly increased as a result of *B. bassiana* inoculation (Figure 3). Based on the measurement of peak area, the jasmonate area was 2.8 × 10^4^ prior to the fungal treatment and increased rapidly to 6.6 × 10^4^ after the fungi and feeding treatment (2.4-fold, *p* < 0.001).

### 3.3. Total Antioxidant Capacity of Plants

**Figure 4:**
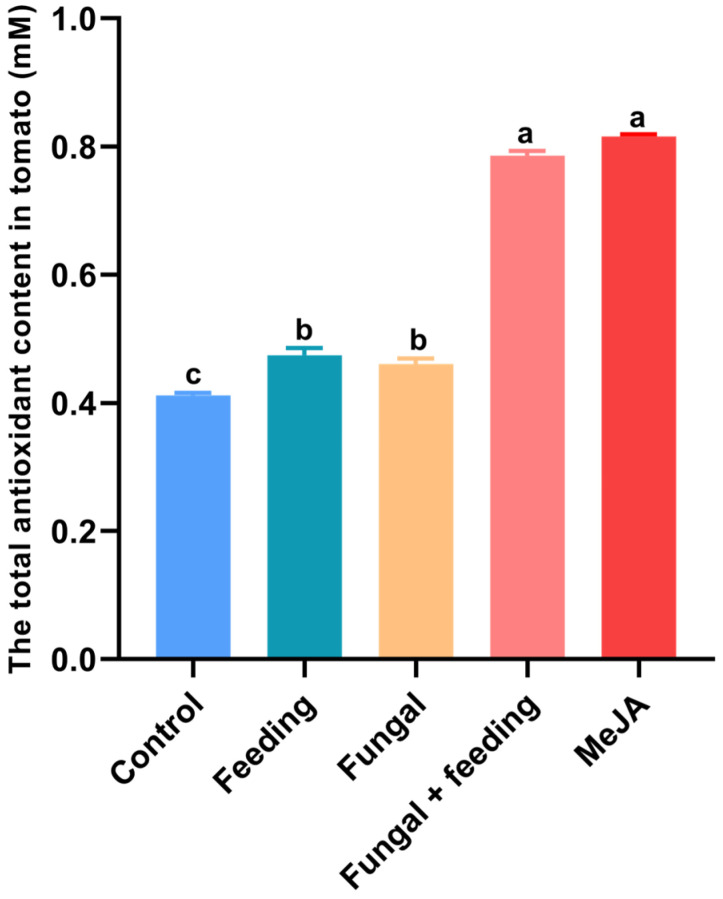
Comparison of tomato plant total antioxidant capacity. Methyl jasmonate (MeJa) means spraying with methyl jasmonate. Fungal + feeding means *B. bassiana* treatment combined with *B. tabaci* feeding. Fungal means *B. bassiana* treatment. Feeding means feeding by *B. tabaci* only. Different letters indicate significant differences (*p* < 0.05; Tukey’s multiple comparison test).

The strength of the antioxidant capacity of plants is directly related to the levels of phenolic metabolites. Inoculation by *B. bassiana* can slightly increase the antioxidant capacity of tomatoes. When the whitefly was feeding on the plant, the antioxidant content increased only to 0.47 ± 0.0115 mM. However, the methyl jasmonate (MeJA) (0.81 ± 0.0036 mM) and *B. bassiana* inoculation combined with pest feeding (0.79 ± 0.0075 mM) significantly enhanced the antioxidant concentrations (F_2,17_ = 6.394, *p* = 0.002, Figure 4), and the staining test of a combination of fungi and insects treating leaves also showed the deepest color (Figure S1A); the antioxidant concentrations of the control groups were only 0.47 ± 0.0115 Mm, and the leaves showed little color (Figure S1C).

## 4. Discussion

External stimulation of plants can induce a high defense gene expression and produce a variety of defensive metabolites, mainly involving phenylpropanoids that mediate the synthesis of phenolic substances, thereby evoking an enhanced response to subsequent pest stress. An activated plant defense system using entomogenous fungi as the stimulus has interesting potential for plant protection [34]. *B. bassiana* and *Metarhizium anisopliae* have also been found to be able to colonize as endophytes in many plant species, such as tomato, corn, and cotton [41]. Over the past few decades, some studies have shown that *B. bassiana* can be artificially introduced into tomato plants by various inoculation methods, such as leaf spraying, root irrigation, stem injection, and seed treatments [42,43,44]. Each of these methods has a different influence on the colonization efficiency of *B. bassiana* in tomato tissues. Our previous research found that when tomatoes were inoculated with *B. bassiana* by leaf spraying, the colonization efficiency reached 100% within 14 days [6]. In recent years, more in-depth insights have been obtained. After the foliar application of *B. bassiana*–GFP, fluorescence was detected in the root and stem tissues of tomatoes [45]. *B. bassiana* was mainly located in the intercellular spaces and vascular tissue [46]. In addition, *B. bassiana* can move within tomato tissues, migrating from the inoculated sites to non-inoculated areas [45,47]. These results suggest that *B. bassiana* can successfully colonize multiple tissues and achieve a systemic distribution in tomato plants.

In this study, we illustrated that *B. bassiana* can act as an exogenous stimulant for tomato plants, activate its own defense system, and even in the mode of pre-induction by the fungus, it can promote plants to respond to subsequent feeding attacks by *B. tabaci*, producing a faster and stronger defensive response. The interaction between *B. bassiana* and tomato plants in our study resulted in the upregulation of the expression patterns of several defense genes, explained to be primarily due to plant recognition of the change in the electrical potential of the plasma membrane [48]. This causes reversible phosphorylation, allowing fast and specific signal transduction of extracellular stimuli to the cytosol and nucleus, namely the mitogen-activated protein kinase (MAPK) cascade [49], an important signaling pathway involved in stress signal transduction in plants.

During the plant defense, the primary signal transduces to the secondary signal process, rapidly increasing the content of the plant-regulating hormone jasmonic acid. This trend was confirmed in our study during both the fungal stimulation and whitefly feeding. Jasmonic acid has been proven to be the most closely related hormone to plant insect resistance [50]. However, plants often use the same defense system when dealing with different stresses. Part of the plant defense intermediates could participate in multiple pathways for insect and microbial resistance [51]. In cotton infected with black spot disease or when fed upon by cotton bollworm, it promotes the increase in chitinase, β-1,3-glucanase, phenylalanine ammonia-lyase, and lipoxygenase enzyme activities [52]. Varying the content of these intermediate products could feed back the signal path in JA [50]. Eventually, this leads to the phosphorylation of transcription factors, which in turn activate an array of defense enzymes (POD, PPO, and PAL) and antioxidant enzymes (CAT, SOD, APx, and GPx) [34,53].

Phenylalanine ammonia lyase (PAL) was highly expressed in our study both as a response to whitefly feeding or following *B. bassiana* treatment. PAL is a key gene, representing an important subset of secondary metabolites in plant defense responses [54]. PAL plays a key role in the resistance to plant diseases. Its synthesis can be enhanced by pathogenic microorganisms as well as some beneficial microorganisms [25,55,56]. Typically, external stimulation leads to plant accumulation of pathogenesis-related (PR) protein, one of the major sources of plant-derived allergens [57]. PAL is more important in the early stages of induction since it is the first enzyme in the phenylpropanoid pathway that leads to the production of phenolic substances [58]. Similarly, the expression of PAL increased significantly during feeding by *B. tabaci*, which has also been reported for feeding by *Phenacoccus peruvianus* on *Bougainvillea* [56]. Phenolic enhancement is regarded as the result of an increase in the activity of PAL in wounded tissues [59].

Phenolic compounds are the largest category of plant-derived secondary metabolites [60], mainly formed via the malonic and shikimic acid pathways. In the shikimic acid pathway, phenylalanine is the most common intermediate for glycolysis and pentose phosphate conversion, being further catalyzed by PAL to form phenolic metabolites [61]. Most phenolic compounds have a crucial role in plant growth and defense against microbes and herbivore attacks. In general, large amounts of phenolic compounds can be detected, and phenylalanine ammonia-lyase activity will significantly increase in the parts of the plant that are subjected to insect feeding [62]. Particularly in plant resistance processes, phenolics have an antioxidant activity as they are hydrogen donors and scavenge reactive oxygen species (ROS) [48]. Here, we showed that the PAL expression level of plants treated by the fungus was the highest, and the phenolic compounds stained the deepest, which was consistent with this statement.

After suffering from insect stress, ROS content in the plant increased rapidly in our study. ROS is an important activator of plant defense. Under normal conditions, the level of cell toxic ROS is kept in homeostasis by effective enzymes and the non-enzymatic antioxidant system [63]. However, under stress conditions, the production of ROS exceeds the plant’s antioxidant capacity, thereby resulting in oxidative stress, which also induces the secretion of antioxidant active substances [64]. Jasmonates, methyl salicylate, and other hormones play a pivotal role as transmission signals in this response to oxidative stress [50,65]. In addition, due to the accumulated level of hormone in plant hosts caused by ROS, higher expression may be induced for a defensive response against external challenges [66].

Based on the above results, our findings have great positive implications for agricultural production. Firstly, understanding the role of *B. bassiana* in activating plant defense mechanisms can offer a new approach to pest management. Instead of relying solely on chemical pesticides, farmers could consider using *B. bassiana* as an alternative or complementary method. For example, in tomato cultivation, introducing *B. bassiana* to the plants could enhance their natural defenses against pests like *B. tabaci* [45]. This could potentially reduce the amount of chemical pesticides used, which is beneficial for both the environment and the cost-effectiveness of farming operations. However, our study currently lacks field experiments, which is a significant limitation. Laboratory and greenhouse studies, while valuable, cannot fully replicate the complex and variable field conditions. In the field, factors such as weather fluctuations, soil heterogeneity, and the presence of other pests and diseases can significantly influence the performance of *B. bassiana* [41].

Field experiments are essential to validate the effectiveness of *B. bassiana* in real-world farming scenarios [67]. They could help determine the optimal application rates, frequencies, and timings for different regions and crop varieties. For example, in different soil types, the colonization ability of *B. bassiana* may vary, which could affect its overall effectiveness in pest control. Field experiments would also provide data on the long-term impacts of *B. bassiana* on soil health, water quality, and biodiversity, which are important considerations for sustainable agriculture [68]. Therefore, future research should also focus on conducting field experiments to further confirm the potential of *B. bassiana*.

## 5. Conclusions

In conclusion, *B. bassiana* can stimulate the synthesis of defense substances in tomato plants, eliciting a response similar to that induced by feeding from *B. tabaci*. However, further studies are required to fully understand the mechanisms of the interaction between entomogenous fungi and plants and their potential for managing the interaction between the pest insect and the plant. Subsequent research can be based on the interaction analysis between pathogen-associated molecular patterns such as chitin and β-glucan and plant pattern-recognition receptors to further clarify the defensive recognition mechanism of plants against entomogenous fungi. Although our study has limitations in terms of this defensive recognition mode, the application potential shown by this approach is still considerable.

## Data Availability

The original contributions presented in the study are included in the article, further inquiries can be directed to the corresponding author.

## Supplementary Materials

The following supporting information can be downloaded at https://www.mdpi.com/article/10.3390/jof11020141/s1, Figure S1: Phenolic substance staining; Table S1: Sequence of primers used in this study.
