# Supplementary material for: Beauveria bassiana Induces Strong Defense and Increases Resistance in Tomato to Bemisia tabaci
Source: J Fungi (Basel). 2025 Feb 13;11(2):141. doi: 10.3390/jof11020141 (PMC11856042; doi:10.3390/jof11020141)
Supplement: Supplementary file 1 [file jof-11-00141-s001.zip › jof-3455477-supplementary.pdf]

Supplementary Materials

Table S1. Sequence of primers used in this study.

Gene	Forward primer	Reverse primer
β-actin	TTGCCGCATGCCATTCT	TCGGTGAGGATATTCATCAGGTT
PAL	TTCAAGGCTACTCTGGC	CAAGCCATTGTGGAGAT
LOX	GGCTTGCTTTACTCCTGGTC	AAATCAAAGCGCCAGTTCTT
PR-1	ACTCAAGTAGTCTGGCGCAACTCA	AGTAAGGACGTTGTCCGATCCAGT
PR2	AAAACGGAGGCCGACAAG	CGGAGGAGACGTATCAGTGGT
CAT	TGGAAGCCAACTTGTGGTGT	ACTGGGATCAACGGCAAGAG
MPK3	ACAACAGCCTCTGGATGAGG	GTTGGATTGAGTGCTATGGCTTC
PBS3	CTTCACATGCTTGGTTATAACTTGC	CGTACCGATCGTGTCATATGAAG
PIN2	GGATTTAGCGGACTTCCTTCTG	ATGCCAAGGCTTGTACTAGAGAATG
SN2	GACTGATCAAGTGAGCAGCAATGC	CTGGCATAGCAAGGGCAAGTC
POX	GCTTTGTCAGGGGTTGTGAT	TGCATCTCTAGCAACCAACG
PPO	CATGCTCTTGATGAGGCGTA	CCATCTATGGAACGGGAAGA

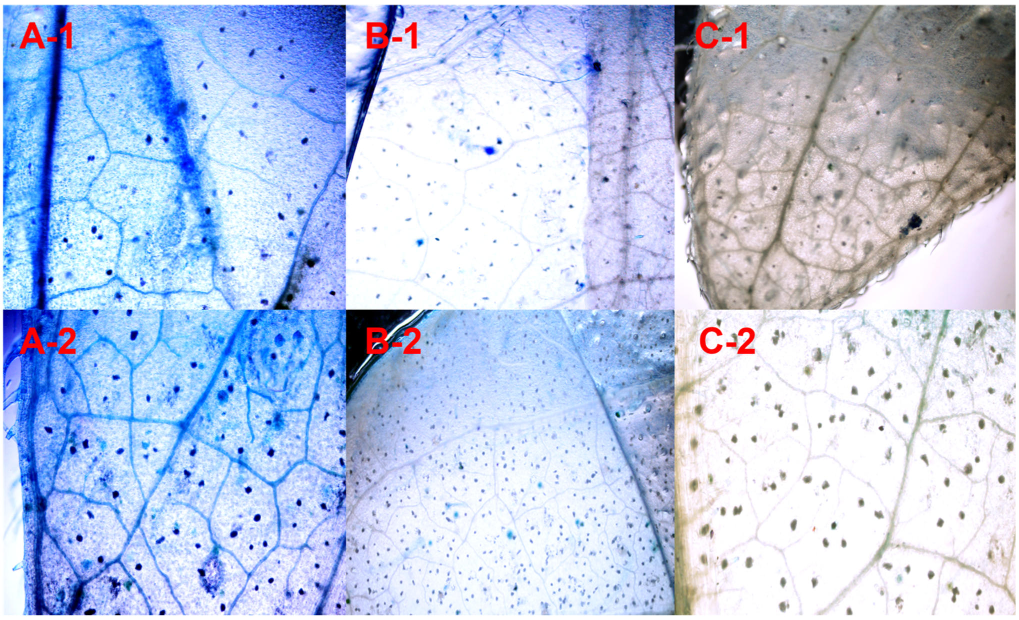

Figure S1. Phenolic substance staining. A A-1 and A-2 are both the phenolic substance staining results of leaves under the treatment of fungal inoculation + B. tabaci feeding. B B-1 and B-2 are both the phenolic substance staining results of leaves under the treatment of B. tabaci feeding. C C-1 and C-2 are both the phenolic substance staining results of normal leaves.
